# Children’s Verbal, Visual and Spatial Processing and Storage Abilities: An Analysis of Verbal Comprehension, Reading, Counting and Mathematics

**DOI:** 10.3389/fpsyg.2021.732182

**Published:** 2021-12-03

**Authors:** Rebecca Gordon, James H. Smith-Spark, Elizabeth J. Newton, Lucy A. Henry

**Affiliations:** ^1^Department of Psychology and Human Development, UCL Institute of Education, University College London, London, United Kingdom; ^2^Division of Psychology, London South Bank University, London, United Kingdom; ^3^Division of Language and Communication Science, City University of London, London, United Kingdom

**Keywords:** working memory, education, visual processing, spatial storage, spatial processing, visual storage, verbal processing, verbal storage

## Abstract

The importance of working memory (WM) in reading and mathematics performance has been widely studied, with recent research examining the components of WM (i.e., storage and processing) and their roles in these educational outcomes. However, the differing relationships between these abilities and the foundational skills involved in the development of reading and mathematics have received less attention. Additionally, the separation of verbal, visual and spatial storage and processing and subsequent links with foundational skills and downstream reading and mathematics has not been widely examined. The current study investigated the separate contributions of processing and storage from verbal, visual and spatial tasks to reading and mathematics, whilst considering influences on the underlying skills of verbal comprehension and counting, respectively. Ninety-two children aged 7- to 8-years were assessed. It was found that verbal comprehension (with some caveats) was predicted by verbal storage and reading was predicted by verbal and spatial storage. Counting was predicted by visual processing and storage, whilst mathematics was related to verbal and spatial storage. We argue that resources for tasks relying on external representations of stimuli related mainly to storage, and were largely verbal and spatial in nature. When a task required internal representation, there was a draw on visual processing and storage abilities. Findings suggest a possible meaningful separability of types of processing. Further investigation of this could lead to the development of an enhanced WM model, which might better inform interventions and reasonable adjustments for children who struggle with reading and mathematics due to WM deficits.

## Introduction

Working memory (WM) is commonly defined as the ability to process information and maintain it for short periods of time, in the pursuit of a known goal (Baddeley and Hitch, 1974; Cowan, 1999; Cowan et al., 2005; Henry et al., 2012). Often separated into verbal WM (i.e., information that can be verbally processed and maintained) and visuospatial WM (i.e., information that is processed and stored in terms of its location and/or visual characteristics), studies have shown that primary school-age children demonstrate marked increases in the quantity of and the length of time that information can be stored in WM. For example, there is evidence that visual WM capacity doubles between the ages of 5years and 10years (Riggs et al., 2006), and the ability to hold verbal information in WM for longer periods of time might be attributed to the emergence of verbal rehearsal in 7- to 8-year-olds (Henry and Millar, 1993; but see Jarrold and Citroën, 2013). Also, results from a study by Gathercole et al. (1994) suggest that the basic structure of WM is evident from 6years of age. Thus, the early to mid-primary school years are an important time of development for this ability.

It is beneficial to briefly explain some key theories of WM, relating specifically to what WM is and what explains individual variation in this ability. First, it is important to consider the enduring multicomponent model of WM (Baddeley and Hitch, 1974). This model consists of a modality-free control system (i.e., the central executive) with two modality-specific subsystems which temporarily store phonological and visuospatial material. Increases in WM ability occur with the use of maintenance strategies which prolong the duration over which information can be maintained. These include verbal rehearsal of phonological information (Baddeley, 1996) and image generation for visuospatial information (Logie, 1995). Second, the time-based resource-sharing (TBRS) model (Barrouillet et al., 2004) argues that an ability to rapidly switch attention between items being processed and items being remembered is fundamental to WM. According to this model, increases in WM capacity are explained by faster processing speeds allowing for more opportunities to refresh items to be remembered. Thirdly, the embedded-process model of WM (Cowan, 1999, 2008; Cowan et al., 2015) sees the role of attention as fundamental to WM capacity. Cowan and colleagues argue that increased, effortful attentional abilities to process salient information is the fundamental component of efficient WM.

Many studies have measured verbal WM and visuospatial WM separately to understand the respective roles in educational outcomes related to mathematics and reading. For example, there is evidence that visuospatial WM is important for mathematics (e.g., Van der Ven et al., 2013; Giofrè et al., 2018; see Allen et al., 2019 for a review) and verbal WM for reading (e.g., Oakhill et al., 2011; Giofrè et al., 2018; see Peng et al., 2018 for a meta-analysis). Verbal WM also shows strong links with word-based mathematics abilities such as problem solving (Rasmussen and Bisanz, 2005; Andersson, 2007; see Peng et al., 2016 for a review) and can be important in the retrieval of mathematics facts from a knowledge base (Gordon et al., 2021). However, studies have also found visuospatial WM to predict reading comprehension in 9- to 12-year-olds (e.g., Pham and Hasson, 2014), suggesting that this type of WM may play a role in reading ability once reading skills have been established. Furthermore, a review by Peng et al. (2016) found mathematics to be related to verbal and visuospatial WM, and to WM tasks that were numerical in nature. Such variability in findings highlights the need for further investigation as to why this might be the case.

A consideration, when investigating relationships between WM and academic outcomes, is the examination of the underlying components of WM to better understand this link. For example, Gordon et al. (2020) examined processing speeds, recall times, processing accuracy and recall accuracy in numerical, verbal and visuospatial WM tasks and found that processing speed and storage in a Counting Span task separately predicted mathematics and reading in 7- and 8-year-olds. More specifically, as manipulations of processing time allowance did not affect storage in WM, faster processing speeds were interpreted as enabling downstream academic abilities rather than increasing WM ability itself. A meta-analysis by Swanson et al. (2009) looked at how storage and processing in short-term memory might explain reading disabilities. They found that poor readers showed deficits in verbal short-term memory tasks that required the recall of digit sequences and phonemes. In addition, it was found that measures combining both storage and processing of digits that were embedded within short sentences also predicted reading ability. Furthermore, a study with primary school children by Gordon et al. (2021) found that the components of WM (i.e., storage and processing) changed in their relationships with mathematics dependent on whether the tasks were verbal or visuospatial in nature. Such findings suggest a possible fractionation of storage and processing within WM in terms of their relationships with educational outcomes. Given this added dimension to the complex relationships between WM and the academic abilities, the current study separately measured storage and processing abilities to better understand how these WM underlying components related to educational outcomes in reading and mathematics.

The conclusions that can be drawn from the literature become more complex when considering the foundational abilities upon which downstream skills, such as reading and mathematics, might rely. Reading can be defined as single word reading of real words often described as ‘word decoding’ or simply ‘decoding’ (Gough and Tunmer, 1986; Hoover and Gough, 1990). It is important to note that this is separate to phonemic decoding which refers specifically to speech sounds and might be measured by the ability to read nonsense words (Van Norman et al., 2018). Verbal comprehension is the ability to understand spoken language, and is a strong predictor of reading ability in children (Reynolds and Turek, 2012). Mathematics can be defined as the “science of structure, order, and relation that has evolved from elemental practices of counting, measuring, and describing the shapes of objects.” (Berggren et al., 2020, webpage). Counting is a method of identifying the number of items in a finite set of those items, and is a strong predictor of mathematics ability (Durand et al., 2005).

There is evidence for the importance of visuospatial WM in reading (Pham and Hasson, 2014) and verbal WM in verbal comprehension (Pham and Hasson, 2014; Schwering and MacDonald, 2020), which in turn predicts later reading ability (Reynolds and Turek, 2012). These findings suggest that verbal WM may better explain verbal comprehension, and visuospatial and verbal WM together explain reading ability, as reading also requires comprehension. Similarly, studies have found that verbal WM predicts broader mathematics ability (Van de Weijer-Bergsma et al., 2015) whereas visuospatial WM predicts counting (Zhang et al., 2014; Georges et al., 2021), which in turn predicts mathematics ability (Durand et al., 2005; Johansson, 2005). These findings showing visuospatial WM to be important for counting, and visuospatial and verbal WM for later general mathematics, suggest that mathematics relies on basic number knowledge (e.g., counting), albeit in a somewhat automated manner. Given this evidence for possible separate roles for verbal and visuospatial WM dependant on whether foundational or downstream abilities are measured, there is a need to further examine the different relationships between these cognitive and educational skills in a single sample. The current study looked at the differing relationships between these four educational outcomes and performance on processing and storage tasks representative of these underlying components of different types of WM.

Whilst many studies have measured verbal and visuospatial WM as two separate abilities, it may be problematic to measure visuospatial WM as a single construct, when, ostensibly, it can be separated into visual and spatial components. This issue was investigated in a review by Allen et al. (2019), with a concluding recommendation that the relationship between mathematics and visuospatial WM could be better understood by examining the subcomponents of the construct. The idea of separating these subcomponents is not new (see Logie and Pearson, 1997; Vicari et al., 2003). In fact, Cornoldi and Vecchi (2003) have proposed a model of visuospatial WM with separate subcomponents specifically for the short-term storage of information related to shapes and colours (i.e., visual WM) and another for the position of objects (i.e., spatial WM). Further, Fanari et al. (2019) examined both visual and spatial WM abilities, finding that they separately predicted mathematics in 6- to 7-year-olds. Specifically, they found evidence suggesting that spatial WM is important in early numeracy, and that both visual and spatial WM predict mathematics as children grow older (but see Vergauwe et al., 2009, that found no dissociation between visual and spatial WM in adults). Finally, a study by Caviola et al. (2020) examined verbal and spatial WM as predictors of mathematics and reading achievement in 7-, 9- and 12-year-olds and found that both verbal and spatial abilities predicted mathematics, whereas only verbal ability predicted reading. Evidently, the separation of visual and spatial abilities may alter the interplay with educational outcomes.

There is value in further examining the separate roles of processing and storage within verbal, visual and spatial WM tasks to better understand which aspects of WM (i.e., processing and storage) enable acquisition of the complex skills of reading and mathematics. Examining how these separate abilities relate to the underlying foundational skills of verbal comprehension and counting can contribute to our understanding of how they, in turn, explain mathematics and reading ability. However, there is a paucity of research that has investigated these separate relationships in a single study. This consideration of the relationships between the components of WM and foundational skills (i.e., counting and verbal comprehension) and the broader abilities of mathematics and reading respectively, could also provide valuable insights into the effectiveness of interventions. These questions are particularly important in relation to the educational outcomes of children in mid-primary education as this is a time when abilities related to increases in WM begin to emerge.

The current study examined the relative contributions of verbal, visual and spatial storage and processing abilities to reading and mathematics in 7- to 8-year-olds, whilst also considering influences on verbal comprehension and counting, respectively. The following research questions were addressed.

1. What are the roles of verbal, visual and spatial storage and processing for reading and mathematics abilities in children aged 7 to 8years?
2. What are the roles of verbal, visual and spatial storage and processing for verbal comprehension and counting in children aged 7 to 8years?
3. Are these relationships different for the foundational skills of comprehension and counting compared the downstream skills of reading and mathematics?

Based on recent research (Gordon et al., 2020), it was predicted that processing abilities would explain individual variation in the downstream skills of reading and mathematics, while storage abilities would explain variance in the foundational skills of verbal comprehension and counting. Specifically, it was predicted that:

1. Spatial storage would explain counting (Zhang et al., 2014; Fanari et al., 2019; Gordon et al., 2020; Georges et al., 2021)
2. Verbal, visual and spatial processing would explain mathematics performance (Van de Weijer-Bergsma et al., 2015; Gordon et al., 2021)
3. Verbal storage would explain verbal comprehension skill (Pham and Hasson, 2014; Schwering and MacDonald, 2020)
4. Verbal processing would explain reading ability (Pham and Hasson, 2014)
5. In addition, although it was expected that visual and/or spatial ability would explain reading, due to a lack of preceding evidence, there were no specific predictions as to which of these abilities might be important for reading

## Materials and Methods

### Participants

An initial sample of 99 7- to 8-year-old children was recruited. As the aim of this research was to assess a representative sample of children in the United Kingdom mainstream education system the only exclusion criterion applied was for children with known developmental delays and/or a Special Educational Needs statement. One child moved to another school before they could complete the third testing session and five more children left school before completing any of the testing sessions. In addition, one child was excluded during their second testing session as it was identified that they were colour-blind and, therefore, unable to complete the spatial processing task. The remaining 92 children (41 male, 51 female) aged between 7 and 8years participated in all testing sessions. All children were unfamiliar with the assessments prior to the commencement of testing. The mathematics curriculum for each school was assessed and it was found that content was marginally inconsistent between schools. This was addressed in the measurement stage and is described in the following Section “Materials.” Mean age and standard deviations at start and end of testing are shown in Table 1.

**Table 1:** Mean age, standard deviation and range at first and last testing session.

Variable (*n*=92; 51 females, 41 males)	Mean	*SD*	Min	Max
Age at testing first session (in months)	93.95	4.23	86	103
Age at testing last session (in months)	97.76	3.55	92	107

### Materials

#### Verbal Storage

Verbal storage (short-term memory) was measured using the digit recall task from the Working Memory Test Battery for Children (WMTB-C; Pickering and Gathercole, 2001). This task was used as it correlates well with word span tasks (Oakhill et al., 2011), yet does not depend on word reading ability. This is important because it avoids the possibility of task impurity in that the task itself overlaps with the core abilities it is attempting to predict (i.e., reading). For the digit span task, the participant was verbally presented with a sequence of digits to be recalled in correct serial order. Digit sequences were designed to appear in random, non-repetitive sequences and were spoken at a rate of one digit per second. With six trials per block, the trials initially consisted of two numbers and increased by one number in each block until the participant was unable to recall four correct trials in a block. Scores for each trial correct were recorded as a value of ‘1’. The sum of these scores denoted the total trials correct as the verbal storage performance index.

#### Verbal Processing

Verbal processing was measured using a time score from one component of the Verbal Inhibition Motor Inhibition (VIMI) task (Henry et al., 2012). The researcher said the words either ‘day’ or ‘night’ out loud and the participant was required to copy by repeating the word. For example:

Researcher: “Day.”

Participant: “Day.”

Researcher: “Day.”

Participant: “Day.”

Researcher: “Night.”

Participant: “Night.”

Researcher: “Day.”

Participant: “Day.”

The time taken to complete the 20 trials was recorded by the researcher using a digital stopwatch. The purpose of this was to record the time taken by each child to process what the researcher had said and then repeat it. Due to the nature of the task, the utterances from the researcher were also included in the time recorded. However, the duration of the words spoken by the researcher were fixed across trials and participants (i.e., spoken immediately after the prior response from the child). Therefore, any delay was due to the hesitancy of the child rather than the researcher. There were twenty trials and the total time taken to complete the task represented verbal processing ability.

#### Spatial Storage

Spatial storage (short-term memory) was measured using the WMTB-C block recall task (Pickering and Gathercole, 2001). For this task, the participant was presented with a plastic tray consisting of an array of nine fixed, three-dimensional cubes. The researcher then pointed to a number of cubes in a sequence and the participant was required to point to each of the cubes indicated by the researcher in the correct serial order. The locations of the cubes were designed to appear in random and non-repetitive sequences. Each block was indicated at a rate of one per second. Trials consisted initially of two items and increased by one number in each block until the participant was unable to recall four correct trials in a block. The scoring was similar to that used in the digit span task, wherein a value of ‘1’ was awarded for each trial correctly recalled. The sum of these scores denoted the total trials correct as the spatial storage performance index.

#### Spatial Processing

Spatial processing was measured by the Colour Number Switch (CNS; Gordon, 2016) task. This assesses each participant’s ability to search for and connect a series of twelve red dots in an irregular pattern across the page. The dots were numbered ‘one’ to ‘twelve’. The time taken on this task was recorded by the experimenter using a digital stopwatch. The time taken on this task denoted the participant’s spatial processing ability.

#### Visual Storage

Visual storage (short-term memory) was measured using the Visual Sequential Memory task from the Test of Memory and Learning (TOMAL; Reynolds and Voress, 2009). The participants were presented with abstract designs in a linear array. They were then required to indicate the order in which they were originally presented when given the same designs in a different order. They did this by pointing at each design and stating the order it appeared in the original presentation (i.e., 1st, 2nd, 3rd, etc.). Up to 12 sets of stimuli were presented, one per page. The first set consisted of two designs. This increased by one on progression to each following set, up to a maximum of 7 designs on the final page. Testing was discontinued if a participant failed to correctly recall the design order in two consecutive trials. The total number of correct positions recalled was recorded.

#### Visual Processing

Visual processing was assessed using a time score from a component of the Odd One Out Span task (Henry, 2001). In this task, the participant was asked to identify, from a horizontal line of three shapes in three separate boxes, which shape was different to the other two (i.e., was the “odd one out”). Two of the shapes were always identical, whilst a third (in any of the three available positions) was the odd one out. The odd one out was always designed to be definitely identifiable without being immediately obvious. For example, two arrows pointing left and one arrow point right; or two squares tilted right and one square tilted left. The time taken on this task was recorded and denoted the participants visual processing ability.

#### Verbal Comprehension

To assess verbal comprehension, a computerised task specifically developed for the study was presented on a Dell 5000 Series Inspiron laptop, and written in E-Prime Version 2.0 (Schneider et al., 2005). The task was driven by a push-button response box operated by the researcher. Children completed a series of twenty trials to calculate their verbal comprehension ability. The participants were requested to complete these trials “as quickly and as carefully as possible.” In individual sessions, each child listened to a sentence (e.g., “Apples have noses”), deciding whether or not it made sense and informing the researcher of their decision by saying “yes” or “no” (in this case, “no”). The researcher recorded the response by pressing the corresponding button on the box. After the twenty trials, the program calculated each participant’s mean verbal comprehension ability based on their time taken to engage in the processing tasks and provide a response. To ensure children were attending to the stimuli (and therefore comprehending it), an 85% accuracy rate with regard to the veracity of the sentences was required for inclusion in further assessment. This calculation of 85% accuracy was based on the automated OSPAN task developed by Unsworth et al. (2005) to assess WM capacity. It was designed to ensure that participants were attending sufficiently to the stimuli. However, no participant performed below this ability level.

#### Reading Ability

Reading ability was measured using the Word Reading task from The British Ability Scales third edition (BAS III, Elliot and Smith, 2011). The participants were required to read single words that became progressively more difficult to decode. Testing was discontinued after 10 successive reading failures. A single point was awarded for each correctly articulated word.

#### Counting

There was a need to ensure the counting task was sensitive enough to identify differences in ability between children aged 7 to 8years, as they are already proficient in this skill (Simms et al., 2013). Therefore, counting ability was assessed using a component score from the Creature Counting task from the Test of Everyday Attention for Children (TEA-Ch; Manly et al., 2001). The task features nine pages presented in a stimulus booklet. On each page, a picture showed a variable number of “creatures” in a tunnel. Interposed at varying stages between the creatures were arrows either pointing up or down. The participant was asked to count the creatures from the start of the tunnel beginning with number one, and to use the arrows as a trigger to switch the direction of the count (e.g., from counting up to counting down, or vice versa). This requirement to switch from counting up to counting down (and vice versa) introduces a level of difficulty that can identify individual differences in counting ability in this age group (Thompson, 1995). Two practice pages were completed prior to commencing the task in order to establish the participant’s ability to count up and down. Each subsequent page was timed. This task was originally designed to assess the executive skill of task-switching. For that ability, a time and error cost were calculated for each child, to represent an attentional capacity to switch between two rules. Therefore, errors would indicate attentional lapses by ‘losing track’ of counting. As the purpose of the current study was to assess counting only, there was a need to minimise the possibility of confounding measurement with this executive aspect. Therefore, only sets that were counted correctly by the child were included. This was done to isolate the speed with which each child could count up and down, without introducing an index of their ability to switch between rules. A calculation of each child’s time score on correct sets was used to measure counting ability.

#### Mathematical Ability

A review of the mathematics curriculum across the schools involved in the study indicated that learning was not consistent across the schools in terms of curriculum content (e.g., one school included teaching percentages, another school did not). This is because Year 3 was not a mandatory testing year in the United Kingdom at the time of data collection. Therefore, the schools were not required to include specific content in their mathematics curriculum for that year. As this would almost certainly induce performance differences due to variations in exposure to certain topics, it was decided that a standardised mathematics test would not provide the correct insight into ability. However, each school had assessed the children’s mathematics ability using the United Kingdom’s Standard Assessment Tasks (SATs; Kirkup et al., 2005), tailored within each school in consideration of the taught topics for that academic year. Hence it was decided that the SATs scores provided by the school would be the best indication of mathematics ability (for a similar approach see Gathercole and Pickering, 2000; Lépine et al., 2005; St Clair-Thompson and Gathercole, 2006). An equivalency measure of ability between schools is included in the Section “Results.”

### Procedure

Each participant was tested individually in a quiet room at school, during class times in the school day. Due to the number of tests, assessment was carried out over three sessions. Each session lasted between 30 and 45min. Occasionally, it was necessary to break a session into two parts due to interruptions such as break-time, lunch, or non-curriculum-related demands (e.g., school play rehearsal, school photograph). However, on such occasions, the testing session was always completed within a single school day. The tasks were presented in the order shown in Table 2. Counter-balancing was not used as this is not appropriate for studies investigating individual differences (Tolmie et al., 2011). This is due to the fact that counter-balancing creates a confound between order and individual differences as the source of variation. With the exception of the SATs mathematics grades, which were collected from the class teachers at the end of Year Three, the remaining nine tasks were administered throughout the Year Three academic year. There was a mean duration of 4months between first and last session.

**Table 2:** Sequence of tasks within each testing session.

Session	Ability
One	Counting
	Verbal storage
	Spatial storage
Two	Reading
	Spatial processing
	Visual processing
	Verbal comprehension
Three	Verbal processing
	Visual storage

## Results

Exploratory analysis identified some skewed distributions for some of the variables. For these variables, the values were converted to z-scores to identify any values more than 2.5 standard deviations from the mean. The corresponding true values were winsorized and substituted with the closest criterion value that fell within 2.5 standard deviations from the mean. This process was undertaken to remove the influence of any extreme responses as recommended by Ratcliff (1993; for a similar approach, see Bayliss et al., 2003, 2005, and Gordon et al., 2020). Means and standard deviations for all measures of storage, processing, verbal comprehension, counting, reading, and mathematics, including the number of values winsorized for each measure are included in Table 3.

**Table 3:** Mean and standard deviations for all cognitive and academic measures.

Task	Mean	*SD*	Min	Max	Values winsorized
Mathematics^1^	8.26	1.34	6	11	1^a^
Reading^2^	67.37	8.1	47	80	2^b^
Verbal comprehension (s)	3.04	1.6	0.89	7.07	2^a^
Counting ability (s)	123.85	37.33	45	202	1^a^
Verbal storage (TTC)	28.98	3.53	22	37	3^a^
Verbal processing (s)	33.65	3.77	24	43	1^a^
Visual storage (TTC)	18.54	4.3	8	28	0
Visual processing (s)	3.32	2.07	0.89	12.9	1^a^
Spatial storage (TTC)	24.26	3.02	17	31	0
Spatial processing (s)	21.23	6.43	12	36	4^a^

1 *school grade converted*;

2 *total words correct*;

a *above the mean*;

b *below the mean*.

*s, seconds; TTC, total trials correct*.

To understand the relationships between each of the cognitive measures and the academic measures, a parametric correlation was run. With regard to the inter-correlations between the academic measures, mathematics and reading were significantly correlated (*r*=0.522, *p*<0.001) and counting speed (lower scores indicating faster counting) correlated significantly with both reading (*r*=−0.415, *p*<0.001) and mathematics (*r*=−0.423, *p*<0.001). Verbal comprehension was not significantly associated with reading, counting or mathematics. All correlations between academic and cognitive measures can be seen in Table 4. Verbal comprehension was related to verbal storage only, with slower response times in the verbal comprehension task linked to lower storage scores (indicated by a negative relationship). Reading correlated with both verbal and spatial storage, as did mathematics ability. Counting was negatively correlated with visual and spatial storage, with slower response times in the counting task times linked to lower storage scores. Counting was also correlated with verbal and visual processing. There were no other significant relationships.

**Table 4:** Correlation between all cognitive and academic measures.

	Reading	Verbal comprehension	Counting	Verbal storage	Verbal processing	Visual storage	Visual processing	Spatial storage	Spatial processing
Mathematics	**0.522** ^**^	0.085	**−0.423** ^**^	**0.284** ^**^	−0.002	0.173	−0.188	**0.326** ^**^	−0.186
Reading	–	−0.143	**−0.415** ^**^	**0.320** ^**^	−0.127	0.034	−0.193	**0.293** ^**^	−0.010
Verbal comprehension		–	−0.118	**−0.216** ^*^	−0.155	0.038	0.052	0.056	−0.046
Counting			–	−0.009	**0.312** ^**^	**−0.365** ^**^	**0.313** ^**^	**−0.290** ^**^	0.093
Verbal storage				–	−0.046	0.057	0.065	−0.049	−0.026
Verbal processing					–	**−0.249** ^*^	0.019	**−0.359** ^**^	0.158
Visual storage						–	−0.094	**0.338** ^**^	0.047
Visual processing							–	**−0.211** ^*^	0.060
Spatial storage								–	−0.196

* *p<0.05*;

** *p<0.01*.

Given the difference in curriculum between the two schools that participated in this study, there was a need to ensure equivalency in terms of the relationships between mathematics and the individual cognitive measures. A comparison of values of *r* from the two schools is shown in Table 5. For all but one of the measures, there were no significant differences in the correlations between mathematics grade and each of the cognitive measures. There was a significant difference in the relationship between mathematics ability and verbal storage (*p*=0.047). Therefore, a further correlational analysis was conducted to examine the links between mathematics ability and verbal storage for each school. For one school there was a significant relationship (*r*=0.358, *p*<0.01, *n*=70); whereas, for the other, there was not (*r*=−0.079, *p*=739, *n*=20). Although this non-equivalence is acknowledged, it is possible that the smaller sample (i.e., *n*=20) was too small to detect the effect. As there was a significant correlation in the larger sample (i.e., *n*=70), and the comparison of values of *r* showed borderline significance (i.e., *p*=0.047) it was decided that the two schools could be considered comparable in terms of the relationships between mathematics and the cognitive measures used in this study.

**Table 5:** Comparison of correlations (*r*’s) between school maths grades and cognitive measures in each of the two schools.

Verbal storage	Verbal processing	Visual storage	Visual processing	Spatial storage	Spatial processing
*Z* = −1.673	*Z* = 0.730	*Z* = 0.084	*Z* = −0.528	*Z* = −1.024	*Z* = −1.139
*p* = 0.047	*p* = 0.233	*p* = 0.467	*p* = 0.299	*p* = 0.153	*p* = 0.127

To identify the roles of verbal, visual and spatial storage and processing in verbal comprehension, reading, counting and mathematics, a series of multiple regressions were run to understand the overall relationships between performance on the cognitive and academic measures. The processing and storage measures for verbal, visual and spatial abilities were entered together as predictors and assessed in terms of the variance explained in reading, verbal comprehension, mathematics and counting in turn. Squared semi-partial correlations are included to show the unique contributions from each predictor to the academic outcomes. These are shown in Table 6. For ease of reading, significant values are shown in bold. The models for reading, mathematics and counting were all significant. In terms of individual relationships with the cognitive measures, counting was predicted by visual storage and processing. Mathematics was predicted by verbal and spatial storage. Verbal comprehension was predicted by verbal storage; however, as the overall model was not significant, this is treated with some caution in the discussion. Reading was predicted by verbal and spatial storage. None of the academic skills were predicted by verbal and spatial processing.

**Table 6:** Multiple regressions showing combined predictors of performance on academic measures.

	Overall model	Verbal storage	Verbal processing	Visual storage	Visual processing	Spatial storage	Spatial processing
Mathematics	***F*(6,83)=4.12** ^**^	***t* =3.091** ^**^	*t* =−0.475	*t* =0.427	*t* =−1.392	***t* =0.2.271** ^*^	*t* =−1.119
	**Adjusted *R***^**2**^ **=0.17**	***β*; =0.300**	*β*; =−0.050	*β*; =0.045	*β*; =−0.138	***β*; =0.253**	*β*; =−0.112
*sr* ^2^		**0.089**	0.002	0.002	0.018	**0.048**	0.012
Reading	***F*(6,83)=4.35** ^**^	***t* =3.660** ^***^	*t* =−0.406	*t* =−1.161	*t* =−1.1689	***t* =2.872** ^**^	*t* =0.825
	**Adjusted *R***^**2**^ **=0.18**	***β* =0.353**	*β* =−0.042	*β* =−0.121	*β* =−0.166	***β* =0.318**	*β* =0.082
*sr* ^2^		**0.123**	0.002	0.012	0.026	**0.076**	0.006
Verbal comprehension	*F*(6,81)=1.11	***t* =−2.139** ^*^	*t* =−1.240	*t* =0.169	*t* =0.673	*t* =−0.060	*t* =−0.320
	Adjusted *R*^2^ =0.01	***β* =−0.231**	*β* =−0.145	*β* =0.020	*β* =0.074	*β* =−0.007	*β* =−0.035
*sr* ^2^		**0.052**	0.017	<0.001	0.005	<0.001	0.001
Counting	***F*(6,83)=4.86** ^***^	*t* =−0.042	*t* =1.877	***t* =−2.652** ^*^	***t* =2.759** ^**^	*t* =−0.539	*t* =0.443
	**Adjusted *R***^**2**^ **=0.21**	*β* =−0.004	*β* =0.194	***β* =−0.272**	***β* =0.267**	*β* =−0.059	*β* =0.043
*sr* ^2^		<0.001	0.031	**0.063**	**0.068**	0.003	0.002

* *p<0.05*;

** *p<0.01*;

*** *p<0.001*;

*sr^2^ =squared semi-partial correlations for each predictor against each outcome*.

## Discussion

This study examined the relative contributions of verbal, visual and spatial storage and processing abilities to reading and mathematics, whilst also considering their influences on the underlying skills of verbal comprehension and counting, respectively. The findings are now discussed in the context of the predictions.

The first prediction was that spatial storage would explain variance in counting skill. However, this was not found to be the case, as visual storage and processing were the only measures that predicted counting. Although this finding does not support the suggestion of Fanari et al. (2019) that spatial WM is important in early numeracy, it could explain why studies have found visuospatial abilities to predict counting (Zhang et al., 2014; Georges et al., 2021). The current study separated visual and spatial abilities and storage and processing WM sub-components, which permitted identification of a specific relationship between visual processing and storage and counting in this age group. This approach supports a recommendation by Allen et al. (2019) that the relationship between WM and numeracy could be better understood by separating visual and spatial abilities.

The second prediction was that verbal, visual and spatial processing would be related to mathematics performance. However, contrary to this prediction, it was found that verbal and spatial *storage* were related to mathematics performance. This finding does not support the results of Gordon et al. (2021). They found stronger links between processing times (within WM tasks) and mathematics than between storage measures and mathematics. Gordon et al. concluded that processing abilities explained downstream mathematics outcomes, although, importantly, they used measures of WM that required concurrent processing and storage, and extracted these measures separately from task performance. The findings from the current study suggest that, without the executive load created by the need to process and store information concurrently, the links between processing and academic abilities are lost. There is a view that WM and short-term storage of information simply represent varying grades of executive attentional abilities (see Unsworth and Engle, 2007). Therefore, the current finding that storage, but not processing, abilities explain mathematics outcomes may be due to there being very little executive load in the processing tasks. This suggests that it is the executive element of the processing tasks that relates to mathematics (see Bayliss et al., 2003, for a supporting argument).

The third, fourth and fifth predictions are best discussed together. It was predicted that verbal storage would explain variance in verbal comprehension. This was found to be the case, although the overall model was not significant so this finding should be treated with caution. It suggests that any effect of verbal storage as a predictor was diluted by the presence of the other predictors. However, there is value in further investigation to understand the role verbal storage plays in verbal comprehension. It was also predicted that verbal processing would predict reading, and this relationship was not found. Finally, it was expected that some form of visual/spatial ability would also explain reading and, indeed, it was found that spatial storage predicted reading. These findings, in part, support the supposition that the early ability to store information verbally is a precursor to later reading ability, when the information is presented non-verbally. As stated in the introduction, there is no preceding evidence to direct a detailed prediction here as to whether visual or spatial processing or storage would be important for reading. Although speculative, the current study provides some early evidence for the role of spatial storage in reading.

Explanations for these findings are now discussed in more detail, in the context of the different abilities. Though interpreted with caution, the finding that verbal storage predicted performance on the verbal comprehension measure supports the idea that verbal comprehension requires the online processing of continuous language input. Diamond (2013) notes that storage in WM may underpin comprehension as it is fundamental for understanding input that unfolds over time. As auditory information is the only stimulus provided (i.e., there is no written text), the participant must hold continuous verbal input in mind for long enough to process and understand it.

For reading, the key material is provided in written and spatial form on a page but reading nevertheless requires the continuous processing of meaning from continuous input, as well as keeping track of spatial position on the page. Therefore, the links between reading and both verbal and spatial storage could reflect the need to hold in mind and process key verbal and spatial information during the reading process (Pham and Hasson, 2014). Although the reading task required single word reading, it was developed based on its robust validity in reflecting reading comprehension (Elliot and Smith, 2011); therefore, the extension here to reading comprehension was not considered unreasonable. A further possibility is that there is a specific spatial demand in single word reading, especially for younger readers, as there is a requirement to accurately map the letters to create the correct word. The absence of a relationship with either visual measure is plausible as the visual information is stored externally (i.e., in written form), reducing demands on resources in this domain. This latter finding also suggests that the separation of visual and spatial WM may provide further insights into the importance of these abilities in reading. The finding of relationships between mathematics and verbal and spatial storage supports previous research that has shown both these abilities might be important in mathematics generally (see Andersson, 2007; Peng et al., 2016). However, the absence of any relationships with visual task performance again highlights the value in separating visual and spatial abilities when examining WM.

It was surprising, however, that for verbal comprehension, reading and mathematics, only the storage variables were found to be important, with no relationships found for the processing variables (verbal storage related to verbal comprehension; and verbal *and* spatial storage related to reading and mathematics). Conversely, counting was the only skill that showed any relationship with processing, showing links to visual processing (as well as to visual storage). There are a few possible explanations for this finding. Firstly, the counting task requires an additional visual processing stage prior to task commencement, in contrast to the other skills measures. Words (reading task), sentences (comprehension) and sums (mathematics) are all provided (either verbally or visually) for the child to use in order to complete the task. However, for the counting task, the child is required to translate the creatures into meaningful information (i.e., numbers). Therefore, there is a need for internal visual storage of the count objects along with continual processing (for the purpose of updating) as children progress through the task. Secondly, links between counting and visual storage and processing may indicate that children who were able to use a visual strategy such as a number line, were better at this counting task (see Schneider et al., 2005, for a review). Thirdly, the visual nature of the task (i.e., counting pictures of creatures and using arrows to indicate the task rule) could simply reflect a visual processing ability. Fourthly, and more speculatively, there is a need for conversion to symbolic numbers in counting objects that requires a visual representation (i.e., of the Arabic symbol). For children with established number knowledge, number symbols are automatically brought to mind when saying the number word (Mundy and Gilmore, 2009). This may assist storage, in the same way as spoken and written words have been argued to automatically trigger each other (*cf*. the visual word form area; Dehaene and Cohen, 2011).

One of the important features about these findings, overall, is that the storage and processing tasks for the measures of verbal, visual and spatial abilities all held separate relationships with reading, verbal comprehension, mathematics and counting. These findings will now be considered in the context of the key WM models.

Only one variable, verbal storage, was related to verbal comprehension, suggesting that the embedded process model (Cowan, 1999, 2008; Cowan et al., 2015) might best represent WM in this instance. This model proposes that WM is the use of attention to activate and hold in mind information from long-term memory. This attentional capacity is argued to be capacity-limited and consciously controlled, whilst supported by unconscious automatic processes. Verbal comprehension demands the activation of information from long-term memory (i.e., word meaning) and continuous attention that is updated as new information (i.e., subsequent words in the sentence) is presented. For the task used in the current study, there was also an additional requirement for the child to draw on their knowledge of the world from long-term memory (as well as accessing word meaning), in order to determine the veracity of the sentence and respond accordingly. This proposal is in line with Cowan’s (1999) argument that WM relies on long-term memory to allow new episodic representations to be available for recall.

Similarly, the role of verbal and spatial storage found here in reading ability is best explained by the embedded-process model (e.g., Cowan, 1999), as verbal and spatial storage could reflect an attentional capacity which activates the relevant information (i.e., phonological and graphic word knowledge respectively) from long-term memory in pursuit of the known goal of reading the word out loud correctly. For both reading and verbal comprehension, the absence of a role for processing in contributing to these academic abilities has been explained previously in this section as being the result of a reduced demand on the need to internalise representations.

Links between verbal and spatial storage and the written mathematics task again suggest the embedded-process model (e.g., Cowan, 1999) as the preferred explanation for the role of WM in this ability. In such a task, the processing of information is external (i.e., in written and numerical text). The child must draw on knowledge from long-term memory, even at the most basic level such as recognising the Arabic numeral ‘2’ as representative of a quantity of two. Attention must be focused on the relevant information in order to complete the task in written form and this information can be verbal (e.g., reciting a number) or spatial such as a reliance on a workspace to support a transition from concrete informal knowledge to formal operation (see Holmes et al., 2008).

Counting ability was related to visual storage and processing, and this might be best explained by the TBRS model of WM (Camos and Barrouillet, 2011). It is noted that the combined abilities of processing and storing information reflect the multicomponent model (Baddeley and Hitch, 1974), but a negative relationship between storage and processing in WM tasks would suggest that the greater a child’s capacity for storing visual information, the faster they are at processing numbers. This trade-off between processing and storage is in line with the TBRS model that posits there is a need to rapidly switch attention from processing to storage in order to maintain relevant information when pursuing a known goal. The faster a child’s processing ability, the better able they are to switch attention and thus maintain information for longer periods before it decays. Although it is noted that the processing and storage tasks in the current study were not integrated (i.e., they were not part of the same task, which does place limits on the conclusions), the links between counting and visual processing and storage could imply a greater role for processing beyond that covered by Cowan’s (1999) embedded-process model. Also, no variance in performance on any academic measures was explained by any of the other processing tasks. This suggests there may be some meaningful separability of types of processing, a finding which does not wholly support other studies (e.g., Bayliss et al., 2003) which have argued for domain-general processing in children, as opposed to domain-specific storage. There are presently no models of WM that argue for discrete types of processing (i.e., verbal, visual, spatial). However, findings from a recent study by Alghamdi et al. (2021) suggest that visual processing ability relates only to the development of visual WM and not verbal WM in 5- to 7-year-olds, supporting the suggestion here that types of processing within WM might be discrete. As the Alghamdi et al. study only examined visual processing ability, there is value in further investigating visual, spatial and verbal processing to understand links with the development of the respective storage abilities in WM. This possible enhanced structure of WM could better inform the links between WM and academic outcomes.

The current study provides some insights as to why the literature continues to be so varied, with differing relationships between WM and reading and mathematics found, depending on the different cognitive tasks used. This may reflect a phenomenon similar to that related to the Miyake et al. (2000; Miyake and Friedman, 2012) model of executive function. That is, when different measures are used for (supposedly) the same executive abilities, disparate relationships with academic abilities are found (see Gordon et al., 2018, for a review). This is referred to as the task impurity problem (Burgess, 1997). That is, when participants complete tasks aimed at measuring a specific ability, other cognitive mechanisms are called into play (e.g., verbal ability in a spatial task). This can make it challenging when trying to isolate what aspect of cognitive task performance relates to a specific outcome (e.g., reading or mathematics). The Miyake model does become more stable as its application moves up the age range (Friedman et al., 2016; see Karr et al., 2018, for a review). In terms of child development this makes sense as, early in childhood, children make use of a mass of processes that are, to a large degree, not directed toward specific tasks or contexts. As they become more familiar with external tasks (e.g., reading and mathematics), these processes become more stable and fractionate out to specific types of function as the tasks demand (Best and Miller, 2010).

At present, for young children, it does not seem to be the case that one model can explain how the development of certain academic abilities is supported by WM. Although the embedded-process model (e.g., Cowan, 1999) goes a long way in explaining the four academic abilities included in this study, it is limited in how it might explain the role of processing. Given what we know about neural processes, it is plausible that brain mechanisms differentiate according to different underlying task demands. This, in part, is in line with the findings of Gordon et al. (2020), who found that time-based demands within WM tasks altered relationships with academic measures, whereby links with storage became weaker and links with processing were strengthened. Although the limitations of some of the tasks used in the current study are acknowledged below, there is value in further pursuing the roles of verbal, visual and spatial processing in WM, and how their influence on educational outcomes might change when task demands are manipulated (e.g., time allowed for processing).

It is acknowledged that the choice of mathematics measure in the current study limits findings to very broad ability. There would be benefit in examining these relationships with mathematical subcomponents, such as those used by Gordon et al. (2021; see also Allen et al., 2019) in their developmental investigation into the WM-mathematics relationship. Similarly, it would be informative to apply the method employed in the current study to different age groups to better understand how the relationships examined here change in younger and older children. It must also be noted that the mathematics measure used in the current study was not consistent across the two schools involved. The end of year mathematics grades awarded by the form teachers were used to minimise a risk of findings being confounded by differences in the curriculum between schools. A comparison of the correlations between each of the cognitive measures and the mathematics measure revealed a possible significant difference between the schools with regard to the link with verbal storage. Further analysis indicated that this difference may be negligible. However, it is acknowledged that a consistent mathematics measure for all participants would be preferable. In addition, it is possible that some of the cognitive tasks used could explain some of the links with academic abilities. For example, the fact that the verbal storage task used numbers might explain the link with mathematics. However, set against this, a study by Oakhill et al. (2011) found that the predictive nature of WM tasks did not depend on the processing stimuli being either word- or number-based. This is in line with other studies that have found different processing stimuli in WM do not affect relationships with academic abilities; rather it is the separability of processing and storage skills that explain this link (Bayliss et al., 2003, 2005).

In summary, the current study found that verbal storage was important for verbal comprehension and reading, and spatial storage was additionally important for reading. However, for counting, visual processing and storage both played a role, but only verbal and spatial storage were relevant for mathematics. We have argued that cognitive resources for tasks that did not require internal representations of the stimuli being monitored related mainly to storage, and were largely verbal and spatial in nature. However, when the tasks did not have externally presented representations (i.e., the numbers sequence in counting tasks), there was a draw on visual storage and processing abilities. Additional research could further examine whether there is indeed a difference in cognitive demands for these internalised tasks. Furthermore, investigation into the possible meaningful separability of types of processing could lead to the development of a new or enhanced WM model, which might better inform interventions and reasonable adjustments for children who struggle with reading and mathematics due to WM deficits.

## Data Availability Statement

The raw data supporting the conclusions of this article will be made available by the authors, without undue reservation.

## Ethics Statement

The studies involving human participants were reviewed and approved by London South Bank University, London. Written informed consent to participate in this study was provided by the participants’ legal guardian/next of kin.

## Author Contributions

RG: conception of article, data collection, analysis, and drafting of manuscript. All authors: critical revision of the text and approved the final version of the manuscript.

## Funding

Original data collection was funded by the Institute for Social Science Research (ISSR) at London South Bank University’s Centre for Research in Psychology.

## Conflict of Interest

The authors declare that the research was conducted in the absence of any commercial or financial relationships that could be construed as a potential conflict of interest.

## Publisher’s Note

All claims expressed in this article are solely those of the authors and do not necessarily represent those of their affiliated organizations, or those of the publisher, the editors and the reviewers. Any product that may be evaluated in this article, or claim that may be made by its manufacturer, is not guaranteed or endorsed by the publisher.
